# Supplementary material for: Crystal structure of Zika virus NS5 RNA-dependent RNA polymerase
Source: Nat Commun. 2017 Mar 27;8:14764. doi: 10.1038/ncomms14764 (PMC5378953; doi:10.1038/ncomms14764)
Supplement: Supplementary Information — Supplementary Figure and Supplementary Note. [file ncomms14764-s1.pdf]

Supplementary Note 1 **Codon optimized sequence of ZIKV RdRp**

TACCATGGGAGCTACGAAGCCCCCACGCAAGGATCAGCGTCTTCCCTCGTGA
ACGGGGTTGTTAGACTCCTGTCAAAGCCTTGGGACGTGGTGACTGGAGTTAC
AGGAATAGCCATGACTGACACCACACCATACGGCCAACAAAGAGTCTTCAAA
GAAAAAGTGGACACCAGGGTGCCAGATCCCCAAGAAGGCACTCGCCAGGTA
ATGAACATAGTCTCTTCCTGGCTGTGGAAGGAGCTGGGGAAACGCAAGCGGC
CACGCGTCTGCACCAAAGAAGAGTTTATCAACAAGGTGCGCAGCAATGCAGC
ACTGGGAGCAATATTTGAAGAGGAAAAAGAATGGAAGACGGCTGTGGAAGC
TGTGAATGATCCAAGGTTTTGGGCCCTAGTGGATAGGGAGAGAGAACACCAC
CTGAGAGGAGAGTGTCACAGCTGTGTGTACAACATGATGGGAAAAAGAGAA
AAGAAGCAAGGAGAGTTCGGGAAAGCAAAAGGTAGCCGCGCCATCTGGTAC
ATGTGGTTGGGAGCCAGATTCTTGGAGTTTGAAGCCCTTGGATTCTTGAACGA
GGACCATTGGATGGGAAGAGAAAACTCAGGAGGTGGAGTCGAAGGGTTAGG
ATTGCAAAGACTTGGATACATTCTAGAAGAAATGAATCGGGCACCAGGAGGA
AAGATGTACGCAGATGACACTGCTGGCTGGGACACCCGCATTAGTAAGTTTG
ATCTGGAGAATGAAGCTCTGATTACCAACCAAATGGAGGAAGGGCACAGAA
CTCTGGCGTTGGCCGTGATTAAATACACATACCAAAACAAAGTGGTGAAGGT
TCTCAGACCAGCTGAAGGAGGAAAAACAGTTATGGACATCATTTCAAGACAA
GACCAGAGAGGGAGTGGACAAGTTGTCACTTATGCTCTCAACACATTCACCA
ACTTGGTGGTGCAGCTTATCCGGAACATGGAAGCTGAGGAAGTGTTAGAGAT
GCAAGACTTATGGTTGTTGAGGAAGCCAGAGAAAGTGACCAGATGGTTGCAG
AGCAATGGATGGGATAGACTCAAACGAATGGCGGTCAGTGGAGATGACTGC
GTTGTGAAGCCAATCGATGATAGGTTTGCACATGCCCTCAGGTTCTTGAATGA
CATGGGAAAAGTTAGGAAAGACACACAGGAGTGGAAACCCTCGACTGGATG
GAGCAATTGGGAAGAAGTCCCGTTCTGCTCCCACCACTTCAACAAGCTGTAC
CTCAAGGATGGGAGATCCATTGTGGTCCCTTGCCGCCACCAAGATGAACTGA
TTGGCCGAGCTCGCGTCTCACCAGGGGCAGGATGGAGCATCCGGGAGACTGC
CTGTCTTGCAAAATCATATGCGCAGATGTGGCAGCTCCTTTATTTCCACAGAA
GAGACCTTCGACTGATGGCTAATGCCATTTGCTCGGCTGTGCCAGTTGACTGG
GTACCAACTGGGAGAACCACCTGGTCAATCCATGGAAAGGGAGAATGGATG
ACCACTGAGGACATGCTCATGGTGTGGAATAGAGTGTGGATTGAGGAGAACG
ACCATATGGAGGACAAGACTCCTGTAACAAAATGGACAGACATTCCCTATCT
AGGAAAAAGGGAGGACTTATGGTGTGGATCCCTTATAGGGCACAGACCCCGC
ACCACTTGGGCTGAAAACATCAAAGACACAGTCAACATGGTGCGCAGGATCA
TAGGTGATGAAGAAAAGTACATGGACTATCTATCCACCCAAGTCCGCTACTT
GGGTGAGGAAGGGTCCACACCCGGAGTGTTG

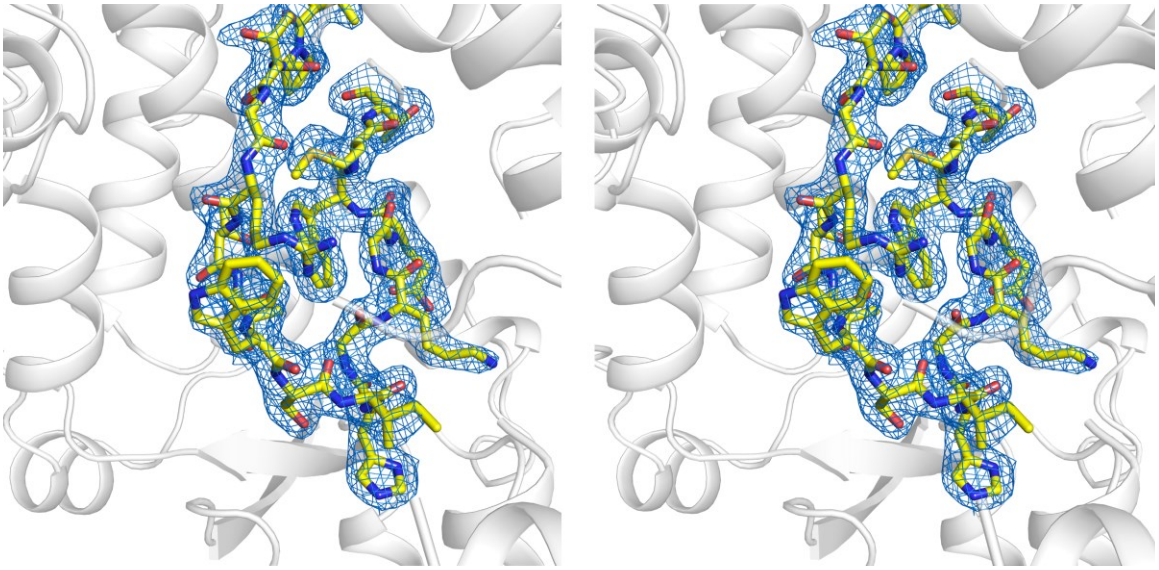

Supplementary Figure 1: Stereo view of the priming loop electron density map ($2F_o - F_c$) of ZIKV NS5 RdRp, with 1.0 sigma.
